# Supplementary material for: Biochemical characterization and inhibition of the alternative oxidase enzyme from the fungal phytopathogen Moniliophthora perniciosa
Source: Commun Biol. 2020 May 25;3:263. doi: 10.1038/s42003-020-0981-6 (PMC7248098; doi:10.1038/s42003-020-0981-6)
Supplement: Supplementary file 1 — Supplementary Information [file 42003_2020_981_MOESM1_ESM.pdf]

Biochemical characterization and inhibition of the alternative oxidase enzyme from the fungal phytopathogen *Moniliophthora perniciosa*

Mario R. O. Barsottini[a,b], Alice Copsey[b], Luke Young[b], Renata M. Baroni[a], Artur T. Cordeiro[c], Gonçalo A. G. Pereira[a,*] and Anthony L. Moore[b,*]

[a] Genomics and bioEnergy Laboratory, Institute of Biology, University of Campinas, Campinas, Brazil

[b] Biochemistry & Biomedicine, School of Life Sciences, University of Sussex, Brighton BN1 9QG, United Kingdom

[c] Brazilian Biosciences National Laboratory, Brazilian Center for Research in Energy and Materials, Campinas, Brazil

[*]Corresponding joint authors: goncalo@unicamp.br (GAGP) and a.l.moore@sussex.ac.uk (ALM)

Supplementary information

Supplementary figures

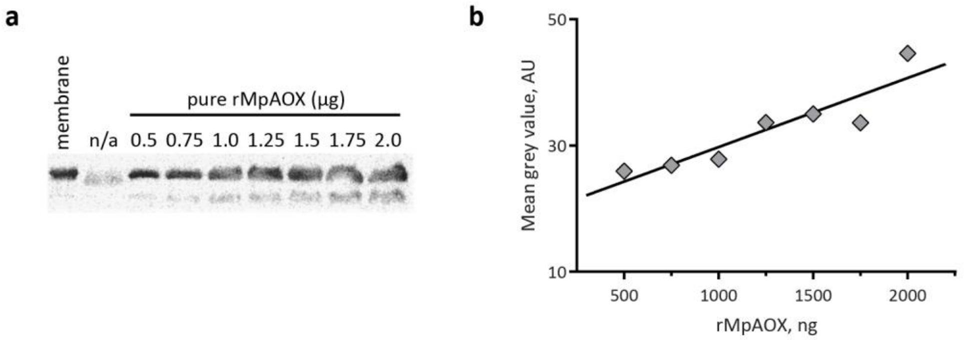

c

Membrane prep	Total protein ($mg\ mL^{-1}$) [a]	AOX ($mg\ mL^{-1}$) [b]	AOX: total protein ratio	O_2 uptake (nmol O_2 min^{-1} mg total ptn^{-1})	AOX specific activity (nmol O_2 min^{-1} mg AOX^{-1})
**A**	2.5	0.77	0.31	577.2	1874
**B**	5.6	0.33	0.06	520.6	8835
**C**	2.1	0.45	0.21	909.6	4245
**Average**				**669.2**	**4,985**

[a] Quantification with the Bradford method.
[b] Quantification through western blot.

Supplementary Fig. 1. Quantification of rMpAOX content in *E. coli* membranes and specific activity calculation. *E. coli* membranes were probed for rMpAOX using the anti-twin-strep antibody and the signal intensity was compared to known amounts of purified rMpAOX. **(a)** Representative western blot image depicting 1 µL of the *E. coli* membrane alongside known amounts of pure rMpAOX. Numbers on top indicate the amount of protein in micrograms. n/a: that sample is of no interest to this work. **(b)** signal intensity of the rMpAOX standard curve, measured as the mean grey value of the corresponding image area. In this case, the membrane signal is 27.1 AU which is equivalent to 767 ng rMpAOX. **(c)** Summary data of three separate *E. coli* membrane preparations. Rates were obtained in the presence of 1 mM GMP.

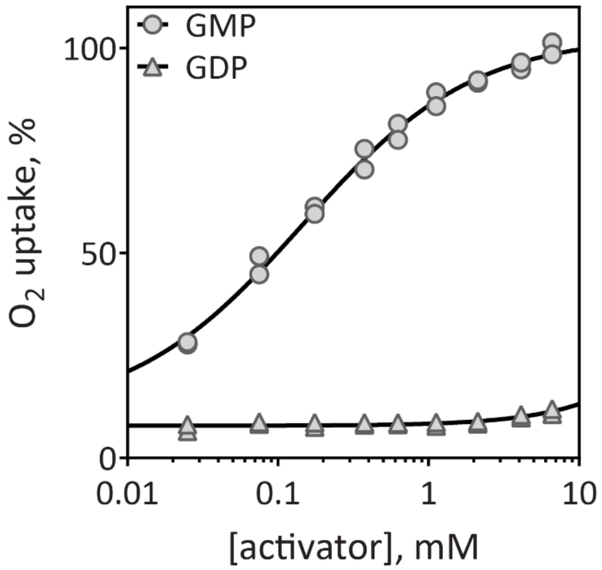

Supplementary Fig. 2. GDP dose-response assay on membrane-bound rMpAOX. GMP data, shown for comparison, is the same as in Fig. 1 in the main text.

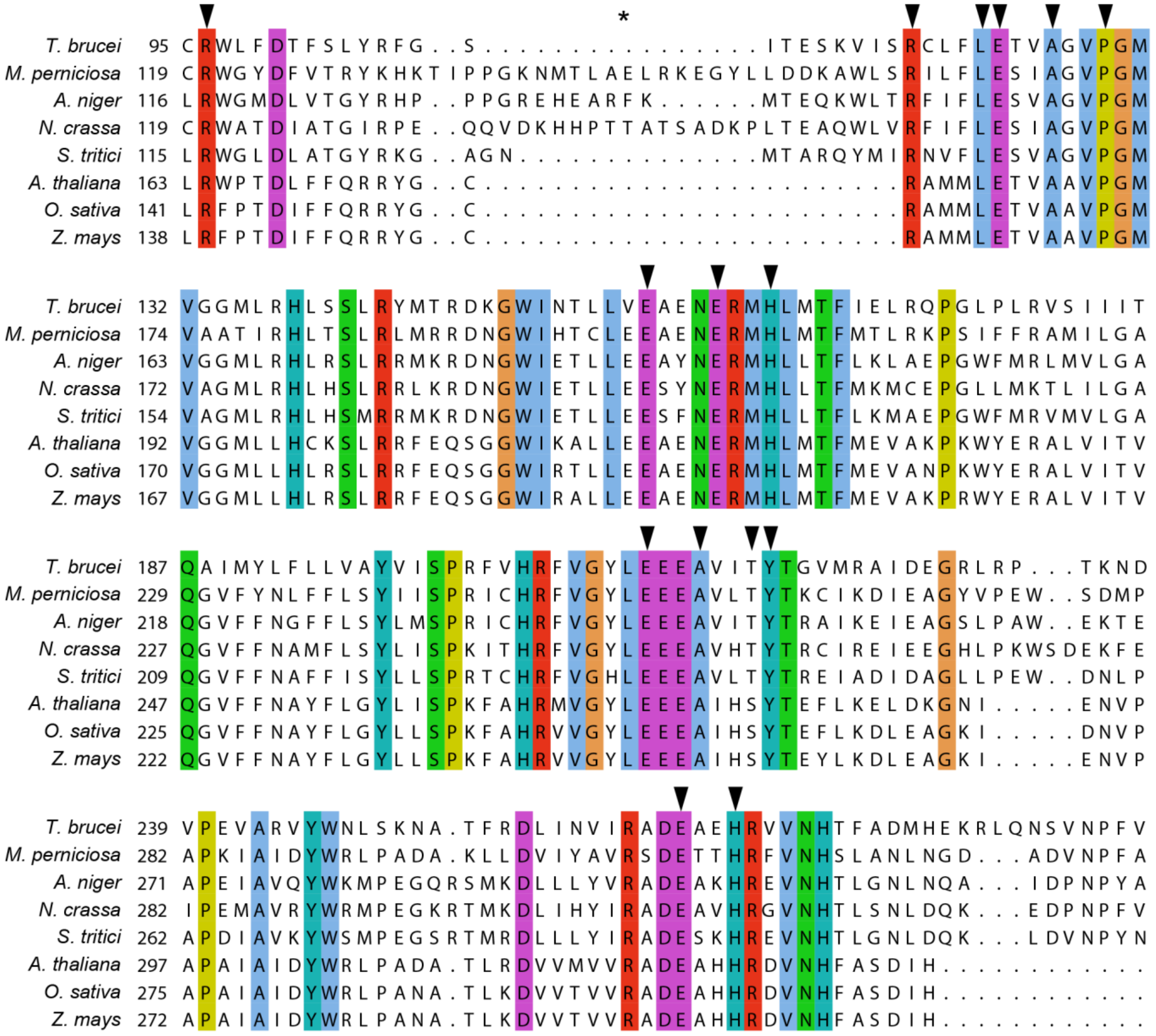

Supplementary Fig. 3. Sequence alignment of AOXs from several organisms. Numbers on the left indicate the corresponding starting amino acid numbering in each row. Arrows show residues important for substrate binding and coordination of the two ferrous ions in the AOX catalytic site, and shaded regions indicate 100% identical residues. Sequences are detailed in the Methods section in the main text.

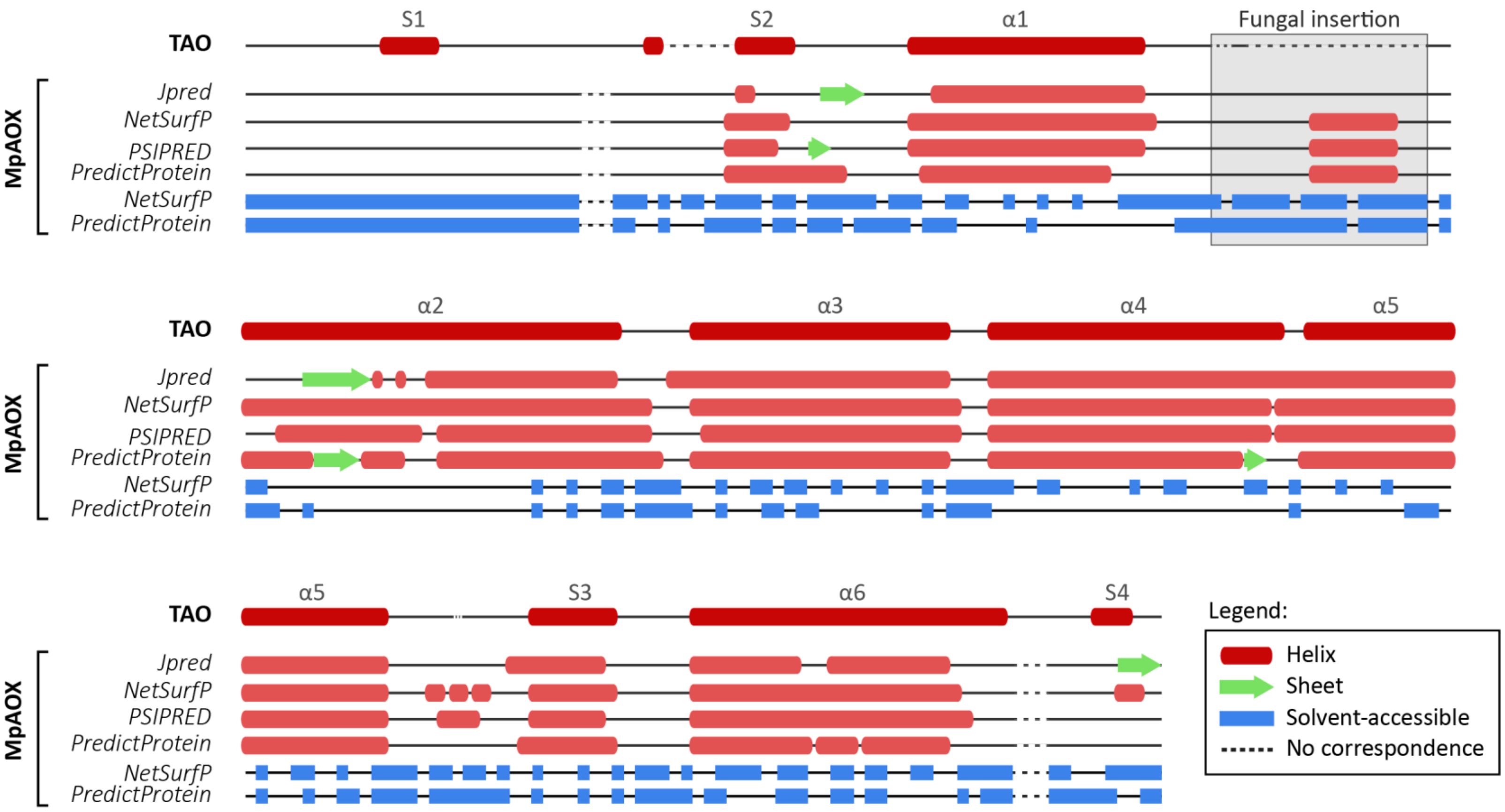

Supplementary Fig. 4. Secondary structure and solvent-accessibility prediction for MpAOX with each line representing one prediction algorithm. TAO is shown at the top for comparison with secondary structure elements as defined in the crystal structure PDB ID 3vv9. Dashed lines depict regions of no correspondence between TAO and MpAOX and the shaded box indicates the 18-residue fungal AOX insertion of MpAOX.

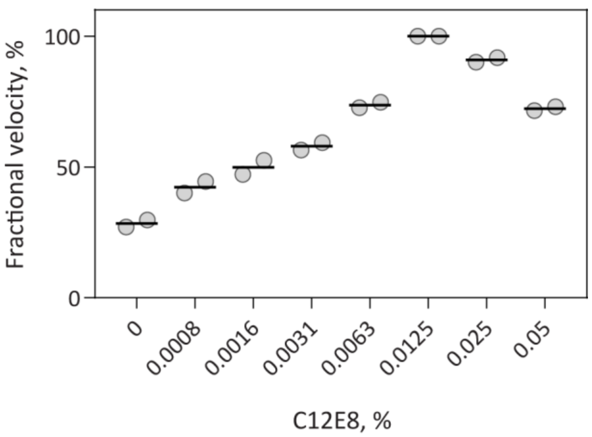

Supplementary Fig. 5. Effect of the detergent C12E8 on purified rMpAOX activity. Mean and standard deviation of two independent replicates.

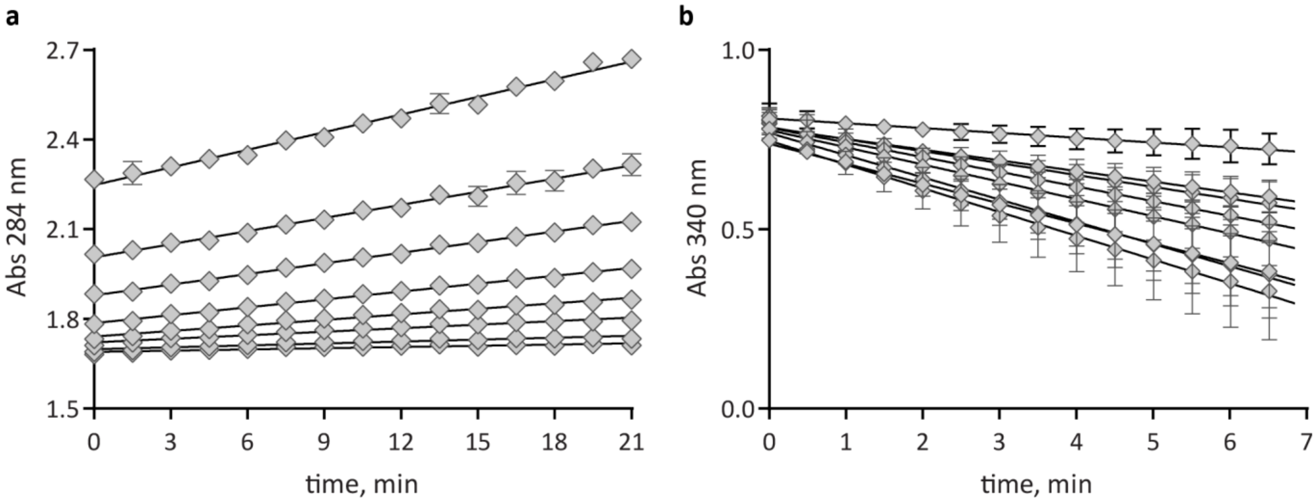

Supplementary Fig. 6. Illustrative progression curves for the enzymatic reactions used for rMpAOX kinetic characterization. **(a)** Solubilised rMpAOX quinol-1 oxidation measurement. **(b)** Proteoliposome NADH oxidation measurement. Symbols represent the mean and standard deviation of technical triplicates at several concentrations of substrate. Fitted lines were used to calculate substrate conversion rate.

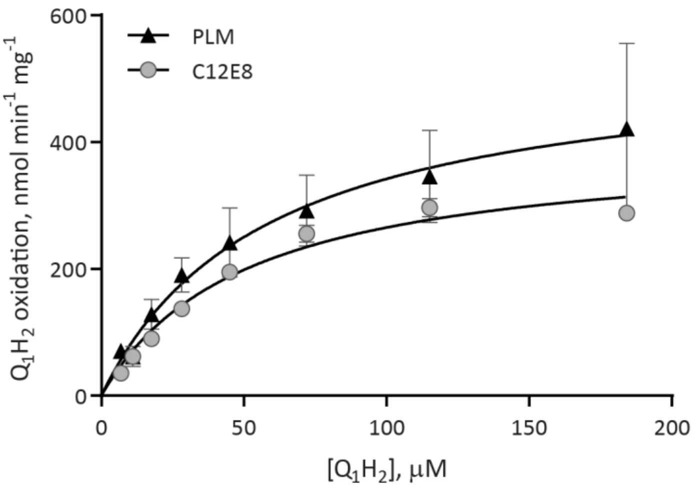

Supplementary Fig. 7. Kinetics of membrane-bound rMpAOX with ubiquinol-1. Proteoliposomes were prepared as described but without addition of NDH-2 and Q_{10}. rMpAOX activity was measured after addition of Q_1H_2 from stock solutions directly into the reaction buffer (black triangles) and soluble rMpAOX in buffer containing C12E8 was used for comparison in side-by-side assays (grey circles). No increase in V_{Max} was observed in proteoliposomes with Q_1, in opposition to assays using Q_{10}. Symbols represent the mean and standard error of two assay and the continuous line is the best fit for the Michaelis-Menten function. It is assumed that Q_1H_2 is evenly distributed in the system.
