# Supplementary material for: Biochemical characterization and inhibition of the alternative oxidase enzyme from the fungal phytopathogen Moniliophthora perniciosa
Source: Commun Biol. 2020 May 25;3:263. doi: 10.1038/s42003-020-0981-6 (PMC7248098; doi:10.1038/s42003-020-0981-6)
Supplement: Supplementary file 2 — Description of Additional Supplementary Items [file 42003_2020_981_MOESM2_ESM.pdf]

Description of additional supplementary items

Supplementary Data 1: Source data underlying graphs and charts in the main figures (Excel file)
